# Periampullary Carcinoma in a 13-Year-Old With Microsatellite Instability Treated Successfully With Pancreaticoduodenectomy

**DOI:** 10.7759/cureus.22139

**Published:** 2022-02-11

**Authors:** Sivaraman Kumarasamy, Lileswar Kaman, Cherring Tandup, Uttam K Thakur, Ajay Savlania

**Affiliations:** 1 General Surgery, Postgraduate Institute of Medical Education and Research, Chandigarh, IND; 2 Surgery, Postgraduate Institute of Medical Education and Research, Chandigarh, IND

**Keywords:** whipple’s procedure, pancreaticoduodenectomy, cancer in young, microsatellite instability (msi), periampullary carcinoma

## Abstract

Periampullary carcinoma in adolescents is very rare and may be associated with hereditary syndromes. Pancreaticoduodenectomy (PD) in adolescents is rarely performed. The experience and results of pancreaticoduodenectomy in adolescents are not well reported. Here, we report a case of periampullary carcinoma, duodenal origin, signet ring type with microsatellite instability (MSI), in a 13-year-old male for which pancreaticoduodenectomy was successfully done.

## Introduction

Periampullary carcinoma is a heterogeneous group of cancers arising around the ampulla of Vater. In adolescent patients, hereditary syndromes such as familial adenomatous polyposis (FAP), Lynch syndrome, Gardner syndrome, and Peutz-Jeghers syndrome should be kept in the differential diagnosis [1,2]. Periampullary cancers requiring pancreaticoduodenectomy (PD) among the adolescent population are very rare; however, it is the only option with pathologies involving periampullary or the head of the pancreas [1,2]. The literature is flooded with studies related to outcomes of PD in the adult population [3]. The reported outcomes of pancreaticoduodenectomy in the adolescent population are comparable with adult populations [1-3]. We present a case of periampullary carcinoma, duodenal origin, in a 13-year-old male for which pancreaticoduodenectomy was successfully done.

## Case presentation

A 13-year-old male presented with obstructive jaundice, abdominal pain, and fever. He had no significant previous surgical and medical history. Family history was not significant. On physical examination, the patient was icteric, and his abdomen showed hepatomegaly with a smooth surface. Blood workup showed elevated total leukocyte count (12000/mm^3^), raised conjugated bilirubin (7.3 mg/dL), and deranged liver function tests (AST = 129 IU/L, ALT = 121 IU/L, ALP = 757 U/L) suggestive of obstructive jaundice. He was treated with intravenous administration of fluids, and piperacillin-tazobactam 4.5 g was administered every eight hourly for a period of seven days. Ultrasound (USG) of the abdomen showed bilobar central and peripheral intrahepatic biliary radicle dilatation (IHBRD) and dilated common bile duct (CBD) with an abrupt cutoff in the distal end.

**Figure 1:**
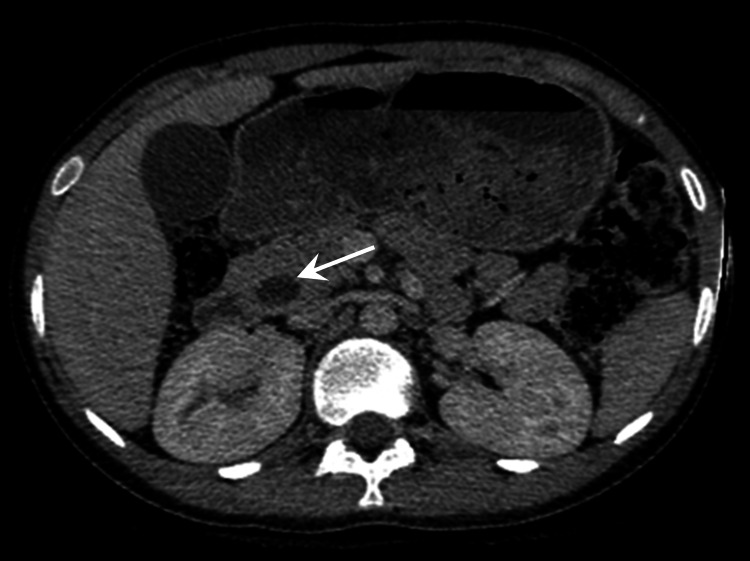
Biphasic CECT of the abdomen showing dilated CBD in its entire course (arrow) with an abrupt cutoff in the distal end before joining the duodenum.

**Figure 2:**
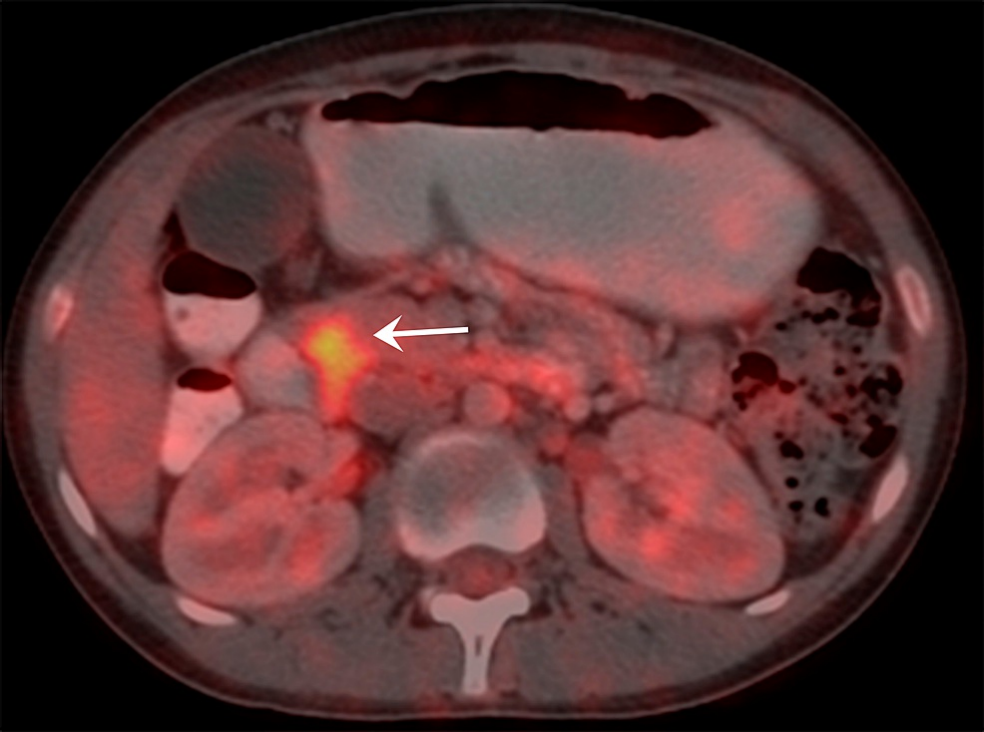
18F-FDG PET-CT showing FDG avid soft tissue periampullary thickening measuring ~2.7 × 1.2 cm with a maximum standard unit value (SUVmax) of 6.9 extending into the D2–D3 junction with ill-defined fat planes with pancreas medially (arrow).

Contrast-enhanced computed tomography (CECT) confirmed the ultrasound findings (Figure 1). There was an ill-defined filling defect in the distal end adjacent to the duodenal opening. Endoscopic retrograde cholangiopancreatography (ERCP) and side view endoscopy showed ulcerative growth around the papilla in the second part of the duodenum (D2). A biopsy was taken from the growth, which was suggestive of adenocarcinoma. A stent was also placed to relieve the cholangitis. Fluorodeoxyglucose PET (FDG PET) showed an FDG avid periampullary thickening at the D2 area (Figure 2). Upper and lower gastrointestinal endoscopy were done to rule out hereditary syndrome, which was normal. Tumor markers CEA (2.7 ng/mL) and CA 19-9 (7 U/mL) were within normal limits.

**Figure 3:**
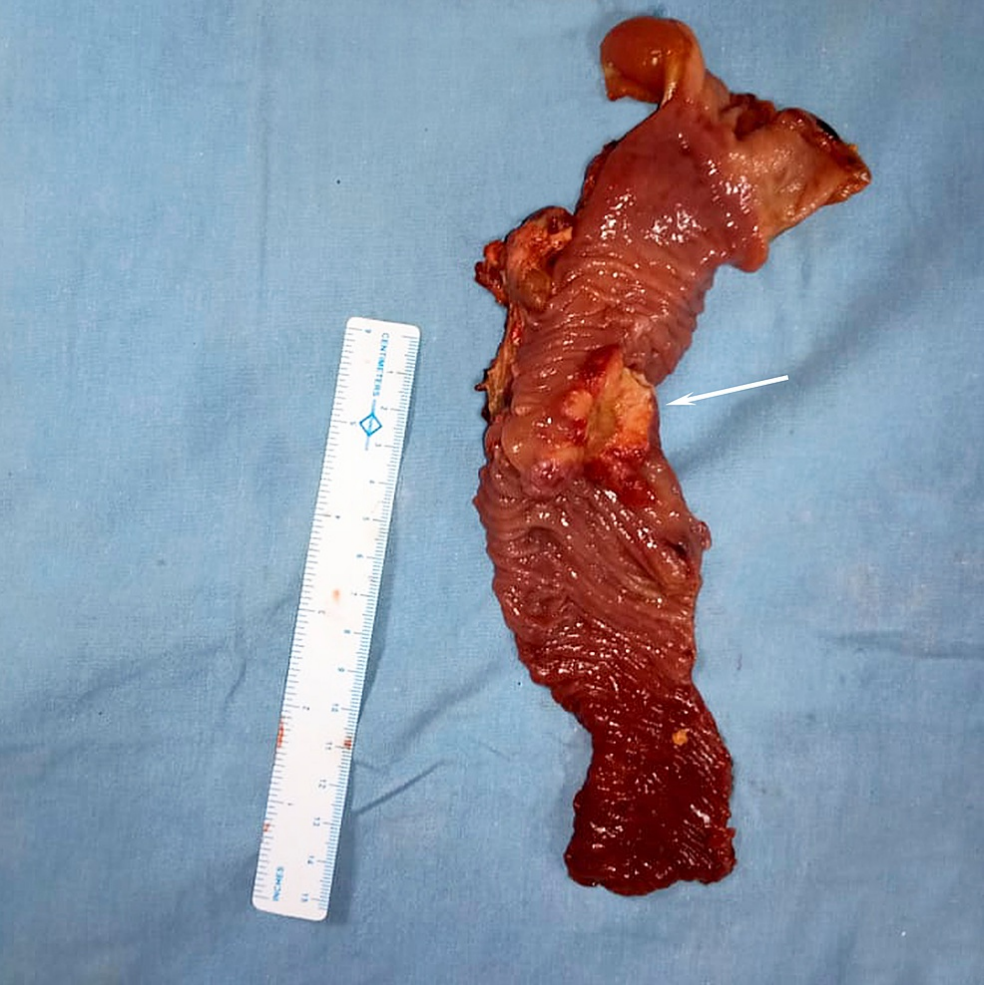
Cut specimen showing a 3.5 × 2.5 cm ulceroproliferative growth in the ampullary region in the D2 (arrow).

After grade II cholangitis was settled, the patient was taken up for Whipple’s pancreaticoduodenectomy. Intraoperatively, there was no evidence of metastasis, and the liver was cholestatic. A 4 × 3 cm hard mass was palpable in the D2 periampullary area. The pancreas was soft, and the main pancreatic duct (MPD) was 3 mm. Duct to mucosa pancreaticojejunostomy anastomosis was done, followed by hepaticojejunostomy and gastrojejunostomy. On the cut section of the specimen, an ulceroproliferative growth was present in the periampullary area (Figure 3). Postoperatively, the patient developed a grade B postoperative pancreatic fistula (POPF), grade B delayed gastric emptying (DGE), and surgical site infection (SSI), for which he was managed conservatively. The patient was discharged on postoperative day 20.

**Figure 4:**
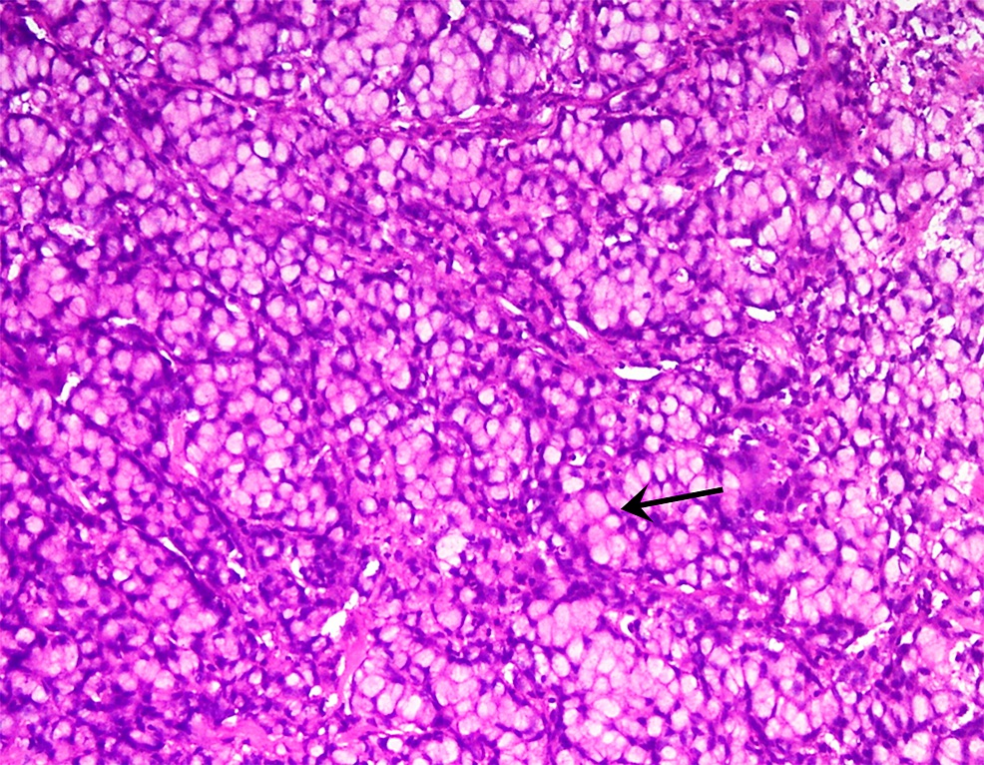
Microscopic examination on high-power magnification showing sheets of signet ring cells seen with clear cytoplasm filled with mucin and eccentric nuclei (arrow).

**Figure 5:**
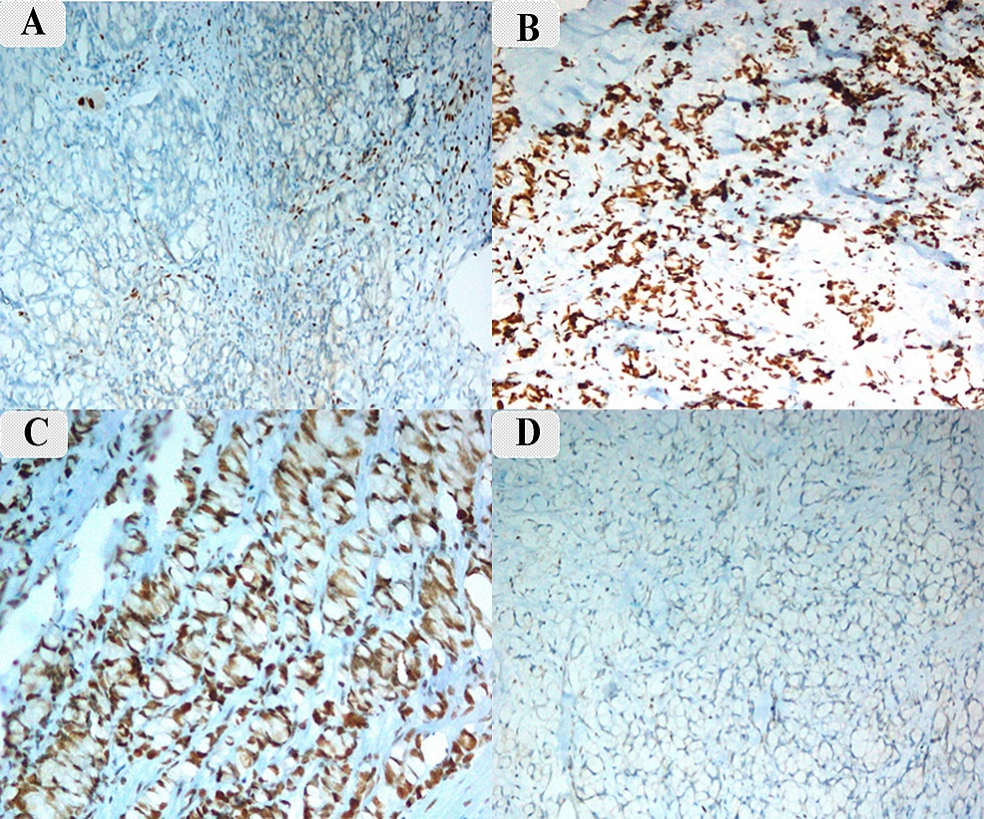
Immunohistochemistry markers. A: MLH1. B: MSH2. C: MSH6. D: PMS2. Tumor cells are negative for MLH1 and PMS2, suggesting a deficiency of MMR proteins (A and D). MSH2 and MSH6 are retained in the tumor cells (B and C).

Histopathological examination demonstrated duodenal adenocarcinoma, signet ring cell type. The tumor was diffusely infiltrating the mucosa and extending up to the submucosa. On a high-power view, there were sheets of signet ring cells (eccentric nuclei and cytoplasm filled with mucin) (Figure 4). On immunohistochemistry (IHC) for mismatch repair (MMR), proteins showed loss of MLH1 and PMS2 (Figure 5A, 5D) with retained MSH2 and MSH6 protein (Figure 5B, 5C) in the tumor cells. The deficiency of MMR protein in this tumor (microsatellite instability (MSI)) was high. The tumor was diagnosed as duodenal adenocarcinoma, signet ring cell type, in an adolescent, probably due to Lynch syndrome (high MSI) or sporadic cancer due to epigenetic silencing. The patient was started on adjuvant therapy and has completed six cycles of injectable gemcitabine. He is doing well on six-month follow-up.

## Discussion

Pancreatic resection in the pediatric, adolescent, and young adult groups is infrequent. Periampullary carcinoma is very rare in adolescents [1,4]. There is no large data available to compute the incidence of periampullary carcinoma in adolescents. In adults, periampullary carcinoma accounts for 20% of all tumors of the extrahepatic biliary tree and 0.5%-2 % of all gastrointestinal malignancies [1-2,4]. Periampullary carcinoma includes adenocarcinoma of the pancreas and tumors of the ampulla of Vater, distal CBD, and duodenum, in which duodenal adenocarcinoma is the least common variety. More than 50% of duodenal adenocarcinoma occurs in the periampullary region. Hereditary syndromes such as familial adenomatous polyposis (FAP), Lynch syndrome, Gardner syndrome, and Peutz-Jeghers syndrome are known risk factors for duodenal adenocarcinoma [2,4]. All patients should be subjected to ultrasound of the abdomen as the first diagnostic test to evaluate the hepatobiliary and pancreatic system. If the ultrasound shows dilated intrahepatic or extrahepatic biliary radicles, cholangiography is required to identify the level of obstruction. Magnetic resonance cholangiopancreatography is a noninvasive modality for cholangiography. ERCP and side view endoscopy can confirm the level of obstruction and can obtain a tissue biopsy for diagnosis and for biliary drainage.

Whipple’s pancreaticoduodenectomy (PD) is a rare procedure in the adolescent population. Therefore, data on postoperative outcomes for this group, including postoperative pancreatic fistula, delayed gastric emptying, and bile leakage, are scarce [3]. Because of the paucity of data, it is difficult to establish the risks and benefits of PD in this age group. PD is a feasible surgical procedure in pediatric malignancies with acceptable morbidity and overall survival [1,2]. Pancreatic leak and pancreatic exocrine insufficiency are the most common short-term and long-term complications, with long-term outcomes depending on the histology of the tumor [4].

Microsatellite instability (MSI) is an important pathogenic mechanism for carcinogenesis in Lynch syndrome [5]. MSI accounts for 15%-20% of periampullary cancers [5]. Lynch syndrome (hereditary nonpolyposis colorectal cancer) is the most common cause of hereditary colorectal cancer, which also predisposes the patients for other cancer, including duodenal and hepatobiliary cancers, especially in young patients [6]. It is due to the inherited mutations of DNA mismatch repair proteins, including high MSI, as in our index case. To make a diagnosis of Lynch syndrome, three steps are essential: fulfillment of the Amsterdam criteria and Bethesda guidelines, tumor testing, and genetic testing [6]. In this index case, there was no family history of cancer in the previous two generations. On tumor testing, he had high MSI, suggestive of DNA mismatch repair. So, he may require genetic testing using multigenic panels to diagnose Lynch syndrome in the absence of clinical criteria. However, due to the high cost and nonavailability of the tests in our institute, we have not got it done. We are also keeping a possibility of sporadic cancer due to epigenetic silencing, which is extremely rare.

## Conclusions

In conclusion, PD is a safe and efficacious procedure for children with pancreatic head and periampullary malignancy. The overall efficacy of surgical treatment along with the reasonably low severity of complications leads us to recommend PD in children when indicated. Young patients diagnosed with periampullary carcinoma should be evaluated thoroughly for hereditary syndromes.
